# Research utilization competency development in the health workforce pipeline: Design and formative evaluation of learning objectives for health professions students

**DOI:** 10.1186/s12961-024-01238-z

**Published:** 2024-12-02

**Authors:** Olive W. Yini Karway, Jeremiah Wleh, Yamah Kpatakolee, Joseph Sieka, Neima Candy, Kristina Talbert-Slagle, Bernice T. Dahn, Wahdae-Mai Harmon-Gray, Laura A. Skrip

**Affiliations:** 1 School of Public Health, College of Health Sciences Campus, University of Liberia, Catholic Junction, 1000-10 Monrovia, Liberia; 2 Yale University, New Haven, CT USA; 3 College of Health Sciences, University of Liberia, Monrovia, Liberia

**Keywords:** Research utilization, Liberia, Health workforce development in LMICs, Competency-driven curriculum development, Evidence consumption, Learning objectives

## Abstract

**Background:**

It is widely recognized that use of research evidence to guide health policy and practice could lead to adoption of life-saving interventions and more effective resource allocation. However, the skills around research utilization are often assumed and rarely taught, particularly in low- and middle-income country contexts. Here we present a set of competency areas and learning objectives developed for institutionalization of research utilization across health professions schools in Liberia. Ahead of implementation and to gauge their perceived value and utility, a participatory formative evaluation was undertaken.

**Methods:**

Focus group discussions were held to gain feedback on a set of research utilization learning objectives and the proposed implementation approach. Focus group participants were drawn from faculty and students at the University of Liberia College of Health Sciences (ULCHS), which houses the country’s only medical and pharmacy schools, along with schools of public health and nursing and midwifery. ULCHS serves an essential role in the health workforce pipeline.

**Results:**

Findings from the focus group discussions identified a limited understanding of research utilization at the ULCHS but a demand for stronger understanding of research methodology and evidence. Participants identified clear examples of how the skills represented in the learning objectives could help specifically their personal careers as well as more broadly the health sector of Liberia. Potential challenges were noted around the incorporation of research utilization learning objectives into existing courses and tended to be logistical (for example, poor internet connectivity and low digital literacy) or around lack of foundational understanding and skills (for example, lack of experience with literature searches and reviews). However, the approach was generally perceived as contextually aware since it would not add new courses, which come with credit fees and extra time commitment, and would focus on practical skills-building rather than theoretical content.

**Conclusions:**

Integrating research utilization learning objectives into existing curricula in health professions schools is expected to enhance uptake and application of research evidence in the Liberian health sector, as students emerge from the workforce pipeline to fill positions in clinical and policy settings. The success of the approach will warrant ongoing evaluation, along with mentorship of faculty, to increasingly incorporate skills and content of local relevance into courses.

**Supplementary Information:**

The online version contains supplementary material available at 10.1186/s12961-024-01238-z.

## Background

Vast amounts of scientific evidence are being generated and published globally, providing scope for increasingly evidence-informed decision-making by policy-makers and others [1]. There is growing recognition among health policy planners that the use of evidence-driven decisions, policies and interventions can support the achievement of global targets to improve health and eradicate diseases [2–5]. The impact of national policies informed by health research has been demonstrated across diverse economic, political and social contexts [6, 7]. Reflecting a long-term, evidence-driven learning process, Mexico’s experience developing and institutionalizing the Popular Health Insurance scheme to improve universal health coverage among the poor has been associated with not only greater coverage of services, but also benefits of these services including reduced maternal mortality and reduced experience of catastrophic healthcare payments [8, 9]. In Cambodia, local generation of evidence to address policy questions contributed to the formulation of Health Equity Funds that have been associated with increased hospital utilization among the poorest segment of the patient population [10, 11]. Despite documented successes around use of evidence-based health policy, the degree of evidence generation, access and translation to policy planning vary greatly across settings [12–14].

In low- and middle-income countries (LMICs), where evidence utilization could lead to more effective allocation of constrained resources, nuanced barriers have challenged efforts to promote use of evidence to guide decisions [3, 15]. In many LMIC settings, sustainable capacity and/or financial support to generate local evidence for addressing policy questions is lacking, even if political will exists for embedding research into the policy process [6, 16–18]. When local evidence or relevant external evidence is available, access may be affected by infrastructural challenges, such as unreliable internet network, or financial constraints, such as affecting ability to access articles that require institutional subscriptions or payment [19]. Another barrier to the translation of health-related research findings to action in LMICs involves difficulty in identifying and accessing relevant consumers of evidence – including policy, programmatic and other non-technical audiences – as well as understanding the capacity and willingness of those consumers to be influenced by evidence [20, 21]. Limited research literacy among potential evidence consumers has implications for their ability to appraise the quality of research and to effectively extract relevant research evidence from its sources to inform health policy decisions [4, 22]. While efforts have been undertaken in developed settings to study mechanisms for motivating organizational shifts towards more evidence-informed decisions [23–25], more foundational skills-building will be critical in LMIC settings to develop the contextually meaningful capacity of health sector evidence consumers to find, understand and evaluate the quality of evidence to inform their decision-making [22, 26].

In the west African country of Liberia, a 14-year civil conflict followed by the 2014–2015 Ebola outbreak devastated the health sector [27, 28]. Post-Ebola recovery efforts for the health sector were largely focused on strengthening the health workforce, improving healthcare delivery infrastructure and building surveillance capacity [29]. However, significant gaps existed between available financial resources and those needed to implement objectives outlined in the Liberia Investment Plan for Building a Resilient Health System [30]. Thus, despite the use of the Ebola experience to guide development of policies and plans, the resilience of Liberia’s health system and its capacity to effectively respond to future shocks remain challenged and potentially inadequate [31]. Reflections on the policy and planning processes in Liberia have suggested that many policy documents exist, but barriers prevent successful implementation [32]. Moreover, despite efforts to engage diverse stakeholders in policy dialogues and decision-making [33], donor priorities have been identified as a driving factor influencing policy development in Liberia [34]. The country has developed a National Research for Health Policy and Strategy (2018–2023). However, little has been documented about the extent to which evidence – such as that which may improve feasibility and sustainability of implementation – presently informs policy-making in Liberia despite limitations in the availability of locally generated research findings being well recognized [35].

To date, there are no public health or basic science Ph.D. programs in Liberia, and while efforts to develop the culture of research are progressively growing, the output by local researchers remains low due to logistical, financial and capacity constraints [36, 37]. The University of Liberia College of Health Sciences (ULCHS) serves as a major pipeline for the health workforce in Liberia, as it houses the only medical and pharmacy schools in the country and the only publicly funded Master’s of Public Health (MPH) program. To systematically bridge foundational skills gaps in Liberia, a research team at ULCHS has chosen the approach of identifying competency areas and designing learning objectives that could be used to capacitate health professions students – future researchers, academics, clinicians and policy-makers of the health sector – with the skills to identify, evaluate and communicate evidence. Here we describe the development process for a set of learning objectives aimed to guide teaching efforts to build contextually relevant research utilization skills across different health cadres. Specifically, we document the methods used to iteratively draft the learning objectives and the results from a qualitative formative evaluation offering feedback on the objectives and the planned process for their introduction into the ULCHS curriculum.

## Methods

### Study site

The ULCHS houses four degree-granting schools for health professions students. These include the School of Public Health (ULSOPH), the School of Pharmacy, the A.M. Dogliotti School of Medicine and the School of Nursing and Midwifery. The University of Liberia School of Nursing and Midwifery is undergoing approval processes and is slated to start in the first semester of the next academic year. In 2022, ULCHS established a Center for Teaching, Learning, and Innovation (CTLI), which is currently overseeing eight activity areas aimed generally at capacity-building within ULCHS and specifically at enhancing research utilization efforts [38].

### Process for drafting ULCHS research utilization competency areas and learning objectives

The process for developing a set of learning objectives involved three groups: (1) ULSOPH faculty who are affiliated with the ULCHS CTLI and responsible for studying how research utilization is taught and learned at ULCHS, (2) MPH students who were brought on as project interns to contribute ideas based on their personal experiences as students in ULCHS and other higher education classrooms, and (3) US-based faculty who serve as mentors to ULCHS CTLI projects on the basis of their extensive experience teaching research utilization at Yale and Vanderbilt Universities. In addition to their mutual interest in and experience with research utilization-related academic activities, the team members also brought individual strengths. The ULSOPH faculty members have diverse research and teaching experience across fields of epidemiology, social and behavioural sciences and biostatistics, as well as professional experiences as clinicians and policy-makers within the Ministry of Health; two faculty members also have extensive qualitative research experience. The three MPH students have undergraduate degrees in Biology, Chemistry and/or Education. They were recruited for this project, in part, due to their experiences as science teachers in primary and secondary schools prior to enrolment in the MPH program and thus their commitment to education and their experience with curriculum development.

The four faculty members from the ULSOPH and three MPH students iteratively met to discuss research utilization (defined in line with Dubrow et al. [39] in terms of the processes of identification, understanding and uptake of scientific evidence for health-related decision-making with recognition of both the evidence and context axes), its relevance in the Liberian health sector and the already ongoing efforts within some ULCHS classes to introduce research utilization. Next, the team conducted a review of existing frameworks for evidence-informed decision-making (EIDM), including the Promoting Action on Research Implementation in Health Services (PARIHS) framework and the World Health Organization’s framework to plan and implement evidence-to-policy processes [40, 41], and literature published on barriers to research utilization in sub-Saharan Africa (for example, [15, 42]).

Based on the background reading and discussion, broad learning outcomes were listed – for instance, “communicating evidence” and “evaluating quality”. Within each of the broad learning outcomes, the specific knowledge and/or skills needed to achieve them were listed. Accordingly, the broad learning outcomes became our competency areas within which specific learning objectives were categorized. There were two guiding principles when preparing the list of requisite knowledge and skills. First, the team focused on learning objectives that did not have dependencies. That is, discrete skills that could be introduced in the context of a class lecture by leveraging assumed pre-existing knowledge were prioritized over more nuanced and highly technical skills that might require entire courses to teach. Second, common situations that health sector players (including health professions schools graduates) often encounter and that could be opportunities for introducing more research utilization were considered. Knowledge and skills of relevance to these situations and aligned with the learning outcomes were captured.

The drafted learning objectives were reviewed, and any additional, “prerequisite” objectives were added. For instance, skills related to “searching for evidence” were added to ensure that students could access the evidence they would then need to communicate to evidence consumers. Once a fully drafted list was developed, the team critically reviewed the list a last time with a focus on ensuring the objectives were clearly stated, measurable and sufficiently specific. The working draft of objectives and competency areas was subsequently shared for feedback from the US-based collaborators.

A list of 14 objectives within six competency areas (Table 1) was thus developed to reflect research utilization skills with relevance to current health sector needs in Liberia. The competency areas ranged from information needs assessment to measurement for the effectiveness of research utilization in that project or decision, and thus were representative of the entire evidence utilization process [39]. The list along with the proposed plan for introducing the learning objectives across existing classes at ULCHS were introduced to faculty and students as part of a formative evaluation process.

**Table 1:** Proposed competency areas and learning objectives for teaching research utilization at ULCHS

Competency area	Learning objectives	Core versus supplementary^*^
1. Information needs assessment	1.1 To engage with stakeholders to identify decisions/policies in the Liberian context which warrant a stronger evidence base	Core
	1.2 To question information that is shared without a reference or health authority (or reputable institution) supporting it	Core
	1.3 To critically review documents/policies/strategic plans during the development phase, such as when invited to provide feedback as part of validation processes	Core
2. Review of available evidence	2.1 To conduct a scientific literature review using an online database	Core
	2.2 To communicate the purpose of a data request and navigate ownership concerns around data access^*^	Supplementary
3. Critical appraisal of evidence	3.1 To define internal and external validity of evidence	Supplementary
	3.2 To apply a checklist for assessing quality of scientific research	Core
	3.3 To be critical of researchers’ methodological decisions	Core
4. Appraisal of evidence consumers	4.1 To identify (situation-specific) evidence consumers	Supplementary
	4.2 To assess the ability to understand evidence (that is, evidence literacy) of different consumer audiences (for example, ordinary citizens, CHWs, doctors, policy-makers, etc.) in Liberia	Core
	4.3 To determine how well evidence consumers can translate the evidence into action/implementation and make use of it	Supplementary
5. Communication of health science knowledge to diverse audiences	5.1 To determine what existing or new channels can be used for communicating evidence to a given audience	Core
	5.2 To establish feedback mechanisms during development (for example, pretesting) and during communication (for example, discussion, engagement factors) of messages to ensure they are effectively conveyed	Core
6. Measurement for the effectiveness of research utilization	6.1 To design tools for measuring effect of influence evidence has on individual-level decision-making	Supplementary
	6.2 To identify approaches for following up with the audience of evidence consumers about how evidence may have influenced their decisions/policies	Supplementary

^*^Added after the focus group discussions per recommendations by participants

### Formative evaluation with focus group discussions

**Fig. 1:**
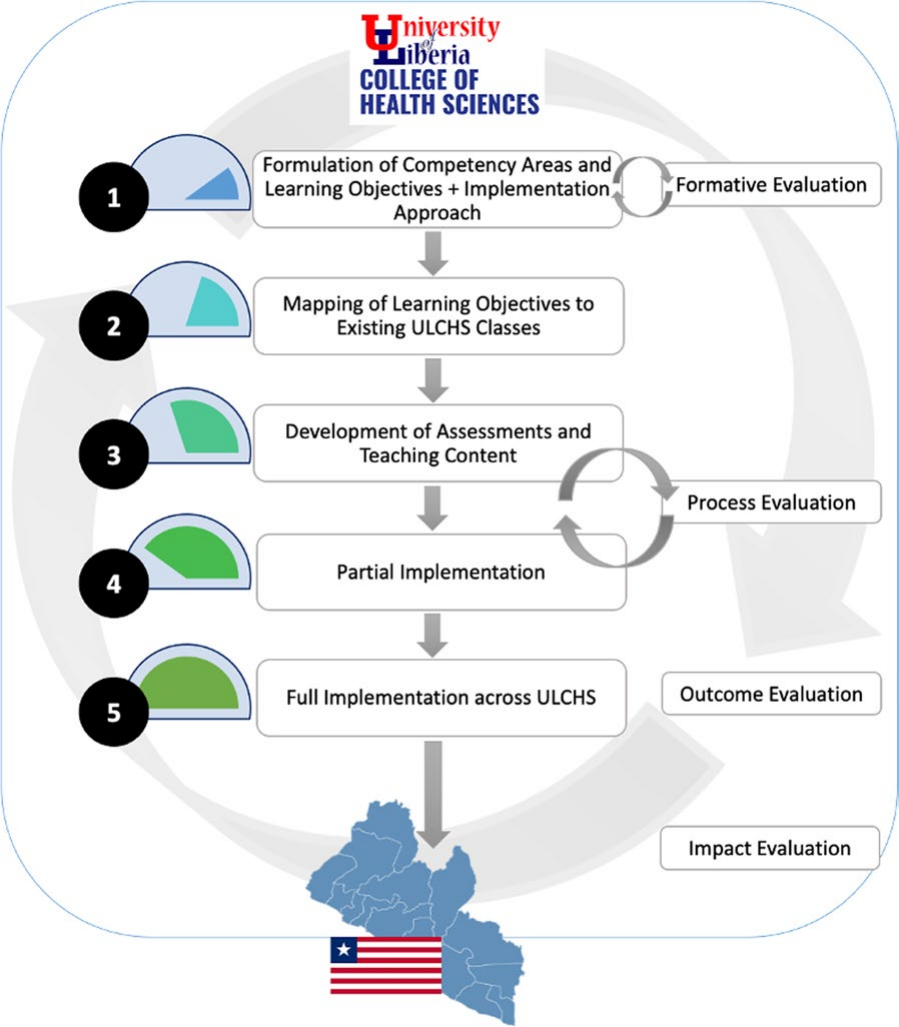
Process of developing, integrating and evaluating research utilization learning objectives as part of health professions curricula. The research utilization learning objectives are intended to be introduced across existing, relevant courses at ULCHS, for example, by modifying or adding case studies, presentations, group work and other practical learning materials with research utilization content and applications. Identification of relevant courses has involved a mapping exercise. During the first semester of the academic year, two classes per ULCHS school will implement the learning objectives (that is, partial implementation), and following a process evaluation to account for lessons learned, full implementation of learning objectives will be undertaken. Checklists and other tools for assessing mastery of research utilization learning objectives will be developed, validated and disseminated

Using focus group discussions (FGDs) with key ULCHS stakeholders, a formative evaluation was undertaken to gather imminent information on the many challenges to improve learning activities. The use of FGDs has been recognized as an effective methodological approach for curriculum evaluation since they offer stakeholders an opportunity to express and then justify their views, requiring more thought and accountability than closed-ended or short-answer surveys, for instance [43]. The team used this method to engage faculty and students in discussions to gain feedback on proposed competency areas and learning objectives for introducing research utilization into the curricula of Schools across the ULCHS. The evaluation also allowed for feedback on the proposed approach to integrating research utilization content. Rather than developing a new course dedicated to research utilization, it is proposed that the learning objectives will be incorporated into existing classes throughout the ULCHS, such as by introducing or augmenting class projects or lectures to reflect relevant content and skills. This approach is intended to reduce the burden on students, who would have to pay for additional credit hours if a new course were added to the curriculum, and to promote sustainability, rather than requiring new, designated personnel to teach research utilization-focused courses. The use of a formative evaluation ahead of implementation of curricular reform was intended to understand and address potential concerns, as well as to serve as the starting point of a continuous feedback process (Fig. 1). Formalized continuous feedback mechanisms have been shown to improve faculty and student satisfaction with curricular changes in health professions programs [44].

*Participants* The study participants were drawn from faculty and students at ULCHS. The college consists of three active schools – the School of Medicine, School of Pharmacy and ULSOPH – and the newly developing School of Nursing and Midwifery. There are approximately 50 faculty members (part-time and full-time) and 450 students on the ULCHS campus.

The target sample size for the focus group study was 30 students (10 from each of the three active schools at ULCHS) and eight faculty members (two from each of the schools, including Nursing and Midwifery). Ultimately, 24 students (14 from the School of Pharmacy, five from ULSOPH and five from the School of Medicine) and four faculty members (two from the School of Pharmacy, one from ULSOPH, one from the School of Medicine and none from Nursing and Midwifery) were recruited. As the Nursing and Midwifery School is still in development, competing priorities around national accreditation activities precluded participation by either of the two active faculty.

Participant recruitment involved a multipronged approach. The study team conducted introductory meetings with the dean or director of each school at ULCHS. At the meetings, the team arranged for one team member to visit seminar classes and inform students about the study. Sign-up sheets were posted in a common space on the ULCHS campus so students who expressed interest could select the focus group day/time that was most convenient for them. Recruitment of student participants specifically targeted full-time ULCHS students who had completed at least one semester of course work. From ULSOPH, participants were recruited from all cohorts. From the School of Medicine, participants were recruited from both clinical and preclinical sections, while the School of Pharmacy participants were recruited from general pharmacy and D-Pharm programs. Nomination of faculty participants was sought from each school’s dean or director, who was asked to recommend two instructors who had work experience in the healthcare sector of Liberia and who had taught for at least one year at ULCHS. The study team subsequently contacted the nominated faculty to schedule their participation in the FGD.

*Data collection* Separate FGDs for ULCHS students and faculty were conducted in December 2022–February 2023. Four student FGDs with 24 participants were held (five participants in each of two FGDs and seven participants in each of two FGDs). One faculty FGD was held with five participants. FGDs lasted between 75–90 min and took place on the ULCHS campus at times that did not conflict with students’ class schedule to remove barriers to participation. The discussions were guided by two sets of questions (specific to students or faculty) to get feedback on the perceived importance of the objectives, anticipated challenges and any recommendations for improvement of the proposed implementation process. The questions used to guide the FGDs are included in the Supplementary Information as Additional File 1.

Three MPH students (authors O.Y.K., J.W. and Y.K.) facilitated the student FGDs, and a faculty member (author L.S.) facilitated the faculty FGD. All facilitators had been involved in the learning objective development phase. Prior to the MPH students’ facilitation of the FGDs, they were trained in administering consent, prompting participants to encourage more discussion as needed and redirecting if participants started digressing from the questions. The training included a mock FGD. Moreover, after the first student FGD, the faculty supervisor conducted a debrief session with student facilitators ahead of additional data collection. The research team used audio recordings via a tablet and a laptop, logged into Zoom, that were placed at different areas in the room where the FGDs were held.

For each student FGD, we aimed to include participants representing at least two schools. The facilitators attempted to ensure that students or faculty members from each of the represented ULCHS schools offered feedback to all questions to encourage different views across schools to be reflected.

*Positionality and reflexivity of the researcher* Given the researchers’ roles as students and professors of the ULSOPH, there may have been a potential risk of selection bias, as the researchers have more interaction with ULSOPH students and faculty than those from other ULCHS schools. To reduce this risk, the researchers designed their recruitment efforts to be transparent and to involve all schools with facilitation by directors/deans.

*Data processing and analysis* Verbatim transcription from the audio recordings was performed. The three MPH student facilitators/researchers (authors O.Y.K., J.W. and Y.K.) transcribed the first FGD from the recording. The three transcriptions were reviewed by the faculty supervisor (author L.S.) and another researcher with qualitative research expertise (author W-M.H-G.). Inconsistency in transcription was discovered to result from segments of the audio recording that were difficult to discern. Those sections were identified and re-transcribed with the recording being played on a device with clearer audio. Each remaining FGD was assigned to one of the student researchers for transcription. A plan was developed for the student researchers to report incidents of unclear audio when transcribing. It was agreed that, when any of the transcribers communicated a challenge in understanding one or more sections of a recording, a second student would be invited to transcribe. The two transcripts would then be reviewed and compared by the faculty supervisor who would determine the final transcript that would be used in the analysis.

Deductive thematic analysis of transcripts was used to explore perceptions of the learning objectives from both those proposed to deliver them (that is, faculty) and those intended to learn them (that is, students). The Framework Method for analysis of the qualitative transcripts was specifically used [45] due to its systematic yet flexible nature to capture themes through collaborative coding [46]. The researchers independently read the transcripts line by line, applying a paraphrase or label (a “code”), to describe what was interpreted in the excerpt of the transcript as important. For the initial transcript, “open coding” was conducted to reflect content of perceived importance from the perspectives of the researchers. All ULCHS researchers, including the MPH students and ULSOPH faculty who had been part of the learning objective development process, were involved in coding. The researchers independently coded the transcripts in Microsoft Excel. After coding the transcript from one student focus group, all researchers met to compare the labels they had applied and agreed on a set of codes to apply to all subsequent student transcripts. Codes were grouped into categories. The process was deductive in that codes were developed for each FGD question, such that the themes were derived from data generated via groups of codes that were deductively generated for each pre-determined area or domain of interest to the research team – for instance, expressions of pre-existing understanding or knowledge around research utilization. The pre-determined domains are included in the Results as Thematic Areas 1–4, with highlighted code groups presented as subthemes per area.

*Ethical considerations *The study protocol and participant information materials were reviewed and approved by the University of Liberia IRB (ULIRB IORG-IRB Number: IRB00013730). Prior to any study-related procedures being conducted, all study participants indicated via writing their agreement to an approved informed consent statement. During the focus groups, participants were encouraged not to use names but rather reference themselves as a student or faculty member of a particular school (Medicine, Pharmacy or Public Health). Furthermore, confidentiality and privacy of information has been protected to the best of the researchers’ ability through removal of identifying information in the transcripts and management of the transcript data on a password-protected device.

## Results

This section presents FGD findings about the approach of introducing research utilization into the curriculum and the contextual relevance of the proposed learning objectives. Findings are organized into four thematic areas on the basis of the FGD questions and subthemes identified as coding groups during the analysis.

### Thematic area 1: existing knowledge of research utilization among ULCHS faculty and students

#### Limited understanding of research utilization

As focus group participants reflected on the proposed incorporation of research utilization into their curricula, most had a general sense of what research utilization is, but their understanding tended to lack depth and nuance. Moreover, participants more often referenced data generation than evidence utilization. Although evidence generation is an integral step for utilization, participants’ limited recognition of research utilization as an independent skillset outside of conducting primary research reinforces the need for these competencies to be taught.

While there is a gap in understanding all dimensions of research utilization, the demand for a stronger understanding of research methodology and evidence was clear. In particular, the need for more research-related activity at the university- and country-levels was emphasized as important for growth.

*“[Teaching Research] will help…by increasing the number of researchers… [without researchers] there will be no new inventions; there will be no new discoveries; we will keep doing the same old thing while others are advancing.”* (Student FGD #3)

### Thematic area 2: perceived usefulness of research utilization

#### Value and utility of research utilization in general and at ULCHS specifically

Participants suggested that, despite ongoing efforts to instill a culture of research at ULCHS, the proposed learning objectives would provide for more practical learning in a setting that relies heavily on theoretical learning. Practical learning was viewed as valuable to understanding and important to finding solutions for problems in the Liberian context.

*“We do not just want to do the theory in class and walk away. If the skill is learned and is not applied, it will be lost.”* (Student FGD #4)

*“Our students [need] to be investigative, generate the evidence and be able to give it out to the relevant stakeholders [as] a way of monitoring the utilization of the policy or its effectiveness.” (Faculty FGD)*

*“There are a lot of problems that we face. It is researchers that tell us what those problems are and find possible solutions, and we want our students to be investigative and find a solution to many problems that we face both in health and across the fields of life.”* (Faculty FGD)

#### Perceived importance of research utilization learning objectives at the individual level

In addition to overall impressions around a systematic introduction of research utilization at ULCHS, participants commented on the importance of specific learning objectives. All proposed objectives were perceived to be relevant/essential to research utilization, but focus group members highlighted the particular importance of individual objectives. They noted how some objectives will provide skills that are important immediately during their academic experience or for their careers.

Several students commented on how the objectives in Competency Area 5, “Communication of health science knowledge to diverse audiences”, would be important to improve their skills for communicating research to people from different educational backgrounds.

*“[The learning objective on] how to assess the ability of people to understand research [Learning Objective 4.2]. So, I think it will be important for me because it is going to help my [research] career... So, if I am given the platform to learn how to communicate research findings to people so that they can understand it irrespective of their social or educational status, I think that will be more effective.”* (Student FGD #3)

*“In terms of communication [Learning Objective 5.2], there is a serious problem, especially in areas where there are health workers. They are ways of presenting information, in terms of talking to your patients. ... So I believe that as health professionals we should have public speaking skills, we should engage ourselves into various writings and we also learn how to interact with people.”* (Student FGD #4)

Others mentioned how the learning objectives around identifying evidence (Competency Area 1) would support them in their clinical decision-making by making them aware of the latest techniques and tools available and would elevate ULCHS students to international standards.

*“As the world changes and the cases that we are having day by day, we need to learn how to search and use relevant evidence to help our patients. Therefore, it will help us to widen our minds and we will learn new things better than just probably learning from the blackboard.” (Student FGD #3)*

*“So now I’m looking at [Learning Objective] 1.1… Conducting a scientific literature review [is very important]... In other countries, other institutions, you see students getting more involved in writing for journals, doing reviews of books etc.”* (Student FGD #3)

Furthermore, understanding evidence and appraising the quality of it (Learning Objective 3.2) or questioning the source of it (Learning Objective 1.2) were considered important for clinical students who are being asked by their communities for advice. One student gave an example of being consulted by community members about a specific set of symptoms and wanting to be able to utilize research to provide the best answers.

*“When it comes to the issue of providing or understanding evidence [to address] certain issues in your community. [For instance,] the other day in my village, people in my group chat said people in the community have sickness and the sickness makes sores. So, they were wondering what was the cause of that thing.”* (Student FGD #2)

Another commented on the perceived importance of Learning Objective 1.2 to evaluate the validity of evidence and its sources.

*“We could be learning things that are not of reality and once we learned thing that is not of reality … it will pollute the community with our wrong information.”* (Student FGD #3)

#### Perceived importance of research utilization learning objectives for the health system

Participants reflected on specific learning objectives that would be important not only for them individually but also for the overall health system. They mentioned how identifying the need for evidence, such as during critical review of documents, policies and guidelines (Learning Objective 1.3), is a fundamental skill needed to promote evidence utilization in the sector.

*“We need to take into consideration all those things like documents, policies, and strategic plans. If you are about to bring a new pharmaceutical product on the market and just go to buy the pharmaceutical product together and route it on the market, it is possible that the pharmaceutical product will be having some efficiency or toxicity problems.”* (Student FGD #2)

In addition to identifying evidence, students expressed the importance at the population-level of proposed learning objectives around monitoring the effectiveness of research utilization once evidence is being communicated and influencing decisions. One student noted the importance around monitoring and evaluating whether research results are being put into practice so that research work can positively impact the population. This process will also provide feedback to researchers on the practical utility of their work.

*“I think it will be important not just to make research but [that] the research we will be conducting, the research we want to do, is something that will be beneficial to us or the people.”* (Student FGD #1)

### Thematic area 3: operationalizing the research utilization learning objectives

Focus group participants commented on how they perceived the approach to implementation and the recommendations they had.

#### Utilizing and expanding existing platforms and resources

Participants reflected on the value of the proposed approach of enhancing existing curricula with research utilization content rather than instituting a new course, due to time and financial constraints.

*“Alright, another thing is the School of Public health has courses that got some of those things under it. So if you want to incorporate it, they should be placed within those courses. You shouldn’t make it as a separate course because there are many credits already. We need a minimum of 52 credits and that is a lot of money we are paying.”* (Student FGD #1)

To ensure that the ULCHS environment overall was enabling for conducting research and learning research utilization, a platform for accessing both online and hard-copy information was suggested.

*“There should be a way that the school can organize an online database for students to do reviews. But I haven’t seen it around here.”* (Student FGD #3)

Several students commented on how information or clinical evidence is being lost in the absence of digital storage, leading to lack of data that could be analysed and lack of evidence that could be utilized in the health sciences.

*“There should be a cloud right where information is being kept. Sometimes most of the hospitals we go to, after two, three years you go back there any record you want you can’t get it. But if it’s being digitized, you know the record will be kept for a long time…and you will be able to get access.”* (Student FGD #1)

#### Feedback mechanisms

Several participants suggested that there should be consistency and quality in delivery of learning objectives. To ensure this, a monitoring and evaluation framework or feedback mechanism was recommended.

*“When these objectives are incorporated into the curriculum, there must be evaluation forms for students to evaluate how well the curriculum has been implemented. With the evaluation forms, students will be able to evaluate the content and delivery of the curriculum.”* (Student FGD #3)

*“I would like for the evaluation of the various instructors to be added too, because sometimes some of these instructors in the school are not very capable... They will just give notes and will not properly explain it to the students… So the evaluation forms will provide insight on who is performing, who is not performing, who is doing well, who is not doing well and it will bring changes too.”* (Student FGD #1)

### Thematic area 4: perceived barriers to implementing research utilization curriculum content in LMIC context

Students and faculty identified barriers that might impact the effectiveness of operationalizing the learning objective framework. Of note, several students identified limited access to computers or other devices and the internet. Even if the barrier of access were to be addressed, participants noted that computer literacy – among faculty in particular – could affect delivery of research utilization content.

*“One of the challenges that I have noticed is the internet services, we don’t have internet services on the campus for students to be able to do their research.”* (Student FGD #1)

*“One challenge has to do with the issue of gadgets and internet support. Many of us students these days we face problem with gadget, like computer or even the android phone. The issue of getting data to run these gadgets even when we have it is another problem and the issue about the entire country internet is another challenge.”* (Student FGD #3)

*“There are people [faculty] who are highly educated but cannot put on the computer.”* (Faculty FGD)

*“I am a student from the School of Pharmacy, one main challenge that we the students will face is that, most of us are not computer literate. Most of us are not people who are knowledgeable about computer usage.”* (Student FGD #1)

In parallel to limited access to evidence due to logistical and computer literacy barriers, participants raised the issue of poor access to data, reports and other evidence due to dynamics around “ownership” and openness of information sharing in the Liberian context. Currently, evidence for utilization is in the form of reports that only certain departments or ministries have access to.

*“The idea of research, if you go to government institutions, to get information is difficult. Take, for example, you go from ministry to ministry, people are not willing to give information. So, there is an information gap. For research information, you need … data for information gathering. So, these are possible challenges that need to be faced.”* (Faculty FGD)

*“One of the challenges is [accessing] resources; what are the [evidence] sources that are available? … Locally, there are a lot of things available. Liberia Medical and Dental Association has published data, NPHIL has published data and LISGIS has published data. But they are not really available [in raw form].”* (Faculty FGD)

## Discussion

Here we present a set of competency areas and learning objectives aimed at ensuring that Liberian students increasingly exit the health workforce education and training pipeline with skills for research utilization. Our process drew upon the research team’s extensive experience in both the health sector and the academic context of Liberia to reflect local needs and scenarios around research utilization. Results from FGDs emphasize the perceived value and utility – at individual and system levels – of skills that health professions students will gain via the planned integration of research utilization competencies. The absence of systematic efforts around teaching research utilization was also evident in that participants indicated that they lacked skills around identifying and communicating evidence, despite recognizing the importance of it. Inconsistent access to the internet and computers as well as gaps in foundational skills among both students and faculty were suggested as challenges to the integration of research utilization at ULCHS.

The barriers identified in this study reflect those that have been noted in other studies focused on research productivity and/or research utilization in the Global South, where researcher-, institutional- and structural-level barriers have been found to be prevalent [18, 47, 48]. The study determined that, at the researcher-level, competing interests on time and poor foundational research-related skills could challenge efforts to teach practical research utilization skills. An overemphasis on teaching versus research in universities paired with lack of investment in research and innovation has been similarly attributed to overly theoretical introductions to research-related skills and low scientific output in Mexico [49]. Other institutional limitations around infrastructural needs (internet and computing resources) and structural issues (lack of open data sources and policies) were noted in this study as well. Reviews conducted by Abouzeid et al. and by Murunga et al. identified similar, multi-level barriers as impediments to development of health research leadership and knowledge transfer capacity-building, respectively, in the Global South [47, 48]. They also emphasized how institutional policies and procedures can be obstructive, which was aligned with comments by FGD participants around the need for effective evaluation procedures at ULCHS to ensure quality teaching of research utilization by faculty that may not have strong research skills themselves.

In response to potential barriers and the recommendations around implementation, the findings presented here have led to concrete changes in the planned implementation of the research utilization competency areas. Of note, the learning objectives were presented to focus group participants as a comprehensive set of skills. However, it was suggested that strengthening the existing foundation for identifying and understanding research evidence may warrant considerable time. The learning objectives deemed as essential were labelled “core”, while some of the more nuanced objectives were labelled as “supplementary” in a revised list of objectives (Table 1). This approach was intended to ensure prioritization of objectives that were foundational to the others, particularly in a setting where instructional time and human resources are constrained. It also allows for the addition of profession-specific, supplementary objectives that may be identified as important for some schools but not all, and thus not warrant attention at the college level. Along these lines, on the basis of feedback from a faculty member, a “supplementary” objective about navigating access to information due to ownership concerns, meaning challenges with limited access to certain data held within ministries or specific Liberian organizations, was added to the original list shared during the focus group (Table 1). Such changes aim to contribute to more enabling and aware procedures as the ULCHS institutionalizes the effort, in contrast to obstructive policies and procedures that have hindered efforts at promoting development of more research-focused environments elsewhere [47, 49].

There is increasing scope for the inclusion of research utilization in health professions education throughout sub-Saharan Africa, where the value of evidence generation and knowledge transfer is being increasingly highlighted as part of the clinical care process and even more broadly as critical for health systems strengthening [26, 50, 51]. A case study from South Africa investigated opportunities for enhancing evidence generation and consumption among undergraduate nursing students through Healey and Jenkin’s Research Teaching and Curriculum Design Nexus [52]. A survey of medical school graduates of Stellenbosch University, South Africa, suggested that a relatively low proportion were prepared with the foundational competencies for conducting and using health systems research, resulting in a call to action to better equip the health workforce with competent scholars who generate, communicate and apply evidence per the university’s policy on graduate attributes for undergraduate students in teaching and learning programmes at the Faculty of Medicine and Health Sciences [53]. The use of a university library for conducting a literature review was part of a more general study to investigate library resource utilization in the Gambia [54]. Utilization of postgraduate students’ thesis research findings in Tanzania [55] as well as students’ demand for and use of local research evidence in Tanzania and Kenya have also been explored through observation studies and without intervention [56, 57]. In Nigeria, studies have suggested that identification and utilization of evidence are lacking not only among university academic staff and governmental education managers but also in the private sector, despite a high volume of evidence being generated and available locally [58]. By documenting a set of proposed competency areas and learning objectives, along with perceptions around their value and utility, the present study holds potential to stimulate more efforts around research utilization as part of the health workforce education and training pipeline.

## Limitations

The FGD results presented here represent evidence as part of a formative evaluation to understand perceptions around research utilization learning objectives and the process of introducing them at ULCHS among students and faculty. Due to the qualitative nature of the study, the results reflect the views of participants and cannot be generalized outside of ULCHS or to faculty and students who did not participate. For the former, future research studying the introduction of research utilization into health professions curricula could include faculty from institutions in other African countries to evaluate how the framework might be applied in diverse contexts. Another limitation was that the faculty from the School of Midwifery and Nursing school were not represented in the FGDs.

## Conclusion

In sub-Saharan Africa, systematic introduction and evaluation of research utilization competencies in health sciences education could facilitate more translation of evidence into action with health impact. Formalizing a set of locally developed, contextually relevant competency areas and learning objectives that could be embedded in curricula for health professions students is one approach to making the research utilization process more integrated into everyday practice for the health workforce. To guide more effective implementation of the curricular changes, the formative evaluation described in this study highlighted the need for a nuanced understanding of research utilization among faculty and students while identifying gaps such as limited competency of staff, difficulty in accessing evidence, poor-to-moderate computer knowledge among students and faculty and other logistical challenges. The approach of training faculty to integrate research utilization learning objectives into existing curricula is intended to moderate some of the barriers but will warrant adaptation to address others. Incorporating research utilization in the ULCHS training program is expected to be an essential pathway to promote evidence utilization at both granular and overarching levels with ultimate impact on the health sector through more informed individual-level decisions and systems-level policies.

## Supplementary Information

Supplementary Material 1.

## Data Availability

All data generated or analysed during this study are included in this published article [and its supplementary information files]. The research utilization competency areas and learning objectives are included in the main text of the manuscript. De-identified transcripts from the focus group discussion are available from the corresponding author upon request.
