# Supplementary material for: Research utilization competency development in the health workforce pipeline: Design and formative evaluation of learning objectives for health professions students
Source: Health Res Policy Syst. 2024 Dec 2;22:158. doi: 10.1186/s12961-024-01238-z (PMC11610060; doi:10.1186/s12961-024-01238-z)
Supplement: Supplementary file 1 — Supplementary Material 1. [file 12961_2024_1238_MOESM1_ESM.docx]

Focus Group Discussion Questions - Student Focus Groups

1. Which of the learning objectives will teach you something new that you think is really important for your professional career? Why?
2. If any of the proposed learning objectives were to be left out, what would students be missing, if anything? That is, how valuable is each objective to your understanding of research utilization?
3. What challenges may students face when learning these competencies within their classes at ULCHS? Please let us know from your perspective as a student/faculty of pharmacy, medicine, or public health.
4. Please describe any challenges that ULCHS will need to overcome in order to introduce new learning objectives into existing classes?
5. Several learning objectives focus on understanding and communicating research findings. How are these related to the content covered in existing classes? Or different from the existing content?
6. The people in this room have many different experiences, both personal and professional. Based on your experiences with the health system in Liberia, are there other skills that you think we should consider adding to increase the use of research evidence in decision-making at the individual level? At the policy-level?
7. Please look at the objectives and suggest edits/changes for improving the program’s learning benefits.

Focus Group Discussion Questions - Faculty Focus Groups

1. Do you think it is important that your students leave their programs at ULCHS with skills to find, share, and use findings from research? If so, why? If not, why?
2. What do you think about having these learning objectives taught as part of health professions curricula at ULCHS? What are advantages or disadvantages to using ULCHS curricula versus in-service learning after graduation? Versus independent learning outside of the classroom for those with interest?
3. What challenges may students face when learning these competencies within their classes at ULCHS? Please let us know from your perspective as a student/faculty of pharmacy, medicine, or public health.
4. Please describe any challenges that ULCHS will need to overcome in order to introduce new learning objectives into existing classes?
5. Several learning objectives focus on understanding and communicating research findings. How are these related to the content covered in existing classes? Or different from the existing content?
6. The people in this room have many different experiences, both personal and professional. Based on your experiences with the health system in Liberia, are there other skills that you think we should consider adding to increase the use of research evidence in decision-making at the individual level? At the policy-level?
7. Please look at the objectives and suggest edits/changes for improving the program’s learning benefits.
